# The Functional Significance of Aposematic Signals: Geographic Variation in the Responses of Widespread Lizard Predators to Colourful Invertebrate Prey

**DOI:** 10.1371/journal.pone.0091777

**Published:** 2014-03-10

**Authors:** Hui-Yun Tseng, Chung-Ping Lin, Jung-Ya Hsu, David A. Pike, Wen-San Huang

**Affiliations:** 1 Department of Life Science, Tunghai University, Taiwan; 2 Department of Biology, National Museum of Natural Science, Taiwan; 3 Department of Life Science, National Taiwan Normal University, Taiwan; 4 School of Marine and Tropical Biology and Centre for Tropical Environmental and Sustainability Science, James Cook University, Townsville, Australia; 5 Department of Life Sciences, National Chung Hsing University, Taiwan; University of Sussex, United Kingdom

## Abstract

Conspicuous colouration can evolve as a primary defence mechanism that advertises unprofitability and discourages predatory attacks. Geographic overlap is a primary determinant of whether individual predators encounter, and thus learn to avoid, such aposematic prey. We experimentally tested whether the conspicuous colouration displayed by Old World pachyrhynchid weevils (*Pachyrhynchus tobafolius* and *Kashotonus multipunctatus*) deters predation by visual predators (Swinhoe’s tree lizard; Agamidae, *Japalura swinhonis*). During staged encounters, sympatric lizards attacked weevils without conspicuous patterns at higher rates than weevils with intact conspicuous patterns, whereas allopatric lizards attacked weevils with intact patterns at higher rates than sympatric lizards. Sympatric lizards also attacked masked weevils at lower rates, suggesting that other attributes of the weevils (size/shape/smell) also facilitate recognition. Allopatric lizards rapidly learned to avoid weevils after only a single encounter, and maintained aversive behaviours for more than three weeks. The imperfect ability of visual predators to recognize potential prey as unpalatable, both in the presence and absence of the aposematic signal, may help explain how diverse forms of mimicry exploit the predator’s visual system to deter predation.

## Introduction

Predators that have the ability to recognize, and subsequently avoid, unprofitable prey will gain fitness advantages. As a consequence, many distasteful or toxic organisms possess conspicuous colour patterns, which can act as a primary defence mechanism by advertising unprofitability to potential predators [1]; this warning advertisement is defined as aposematism. Responsive predators save time and energy through the early detection of unpalatable prey, which increases prey survival [2], [3] and decreases wasted predation attempts by predators [4]. Displaying obvious visual signals is thus an important evolutionary strategy that has evolved independently in a wide range of taxa, such as Lepidoptera (e.g. moths [5]), Coleoptera (e.g. ladybirds [6]), Hemiptera (e.g. true bugs [7], [8]), Squamata (e.g., coral snakes [9]), Anura (e.g., poison frogs [10]) and Teleostei (e.g., catfish [11]). Not all species with bright colouration are aposematic, and in these instances, the colouration serves other important functions, such as prey attraction, mate attraction or assessing competitive ability of rival conspecifics [12], [13], [14]–[16]. Determining the functional significance of bright patterns in diverse animal groups will aid in a fuller understanding of how and why these signals evolve.

Conspicuous colouration and/or patterning is easily detected, learned, and avoided by vertebrate and invertebrate predators [2], [4], [17], both in the laboratory [17]–[21] and in the field [22], [23]. Elements of the conspicuous colouration itself can enhance the cognitive ability of predators in response to unpalatable prey [17], [24]. For example, high chromatic contrast and brightness of aposematic prey not only increase the predator learning speed, but also memory retention of aversive responses [17], [25]. Predators, such as the mantis, can retain aversive responses towards conspicuous unpalatable prey for longer than towards cryptic prey [17]. Memory retention is not only affected by the visual conspicuousness of prey, but also by the interaction between the type of stimulus within a signal (e.g. odour, shape, behaviour) and the strength of the aversion response [26]. These factors can accelerate avoidance learning and memory retention. Because of the complexity of memory formation, the duration of aversion may be variable across predator taxa, and relevant studies of the duration of aposematic prey recognition are scarce [4], [26].

**Figure 1:**
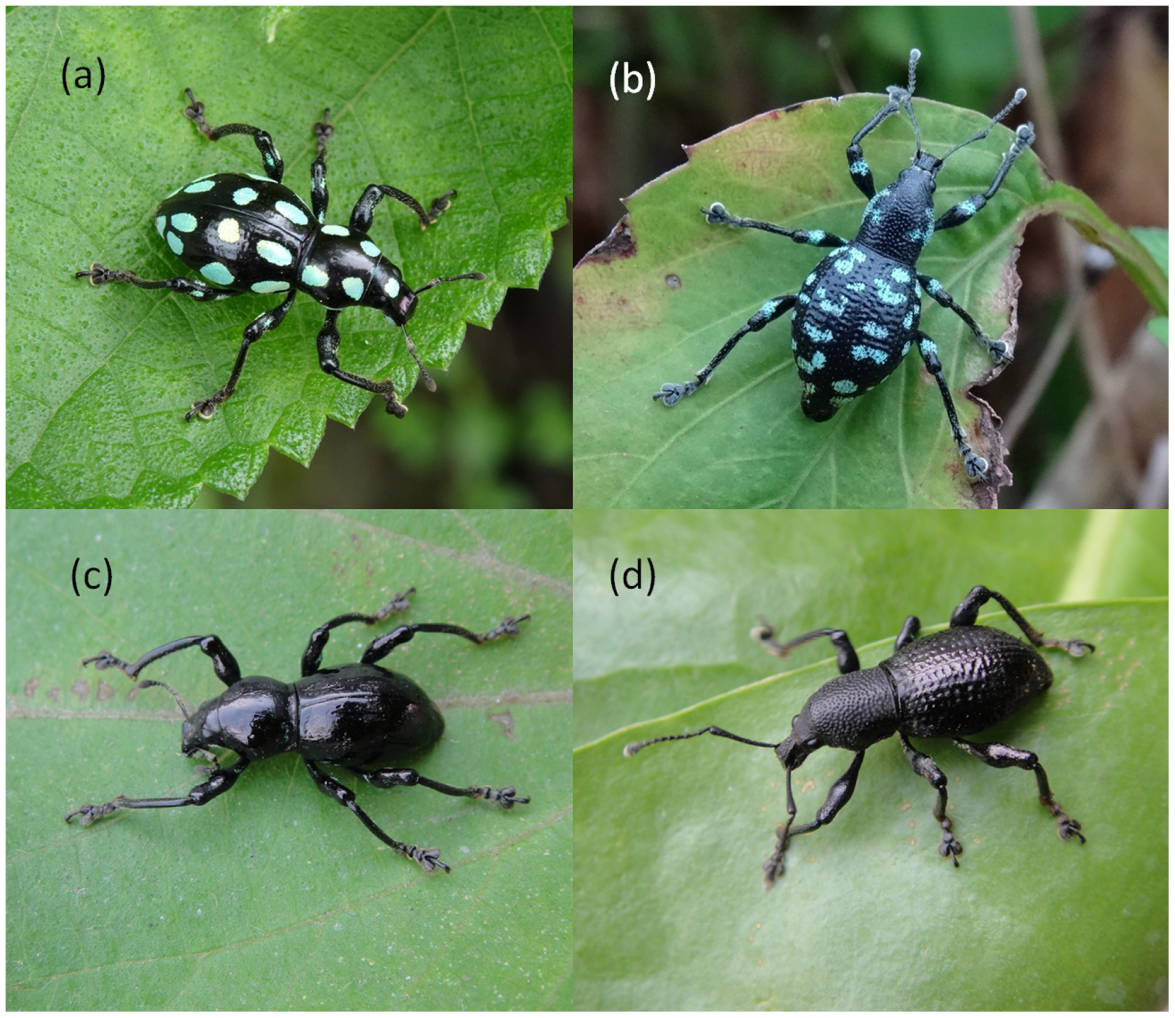
Pachyrhynchid weevils used in our experiments. (a) *Pachyrhynchus tobafolius*; (b) *Kashotonus multipunctatus*; (c) and (d) show *P. tobafolius* and *K. multipunctatus*, respectively, with their aposematic markings experimentally masked with a black marker pen.

Pachyrhynchid weevils (Insecta: Coleoptera: Curculionidae) are perhaps the most colourful and charismatic group of insular beetles found throughout the Old World tropics [27], [28]. Their thoraces, elytra, and legs are often decorated with brightly coloured stripes, circles, and/or spots against high-contrasting dark bodies (Fig. 1). Alfred Russel Wallace first hypothesised that the conspicuous colours of pachyrhynchid weevils served as warning signals to predators [29], [30]. Weevils could be unpalatable for some predators because of their extremely tough exoskeleton (chemical defences are unknown in this group of insects [27], [31]). After more than 120 years, however, we still do not understand the adaptive significance of bright colouration in pachyrhynchid weevils, despite their conspicuous nature and high diversity throughout the Old World tropics. Although these weevils show an astonishing diversity in colouration, their colour structure and iridescence mechanisms are not well understood [29], [32]. Many sympatric insects, comprised of different weevil genera [27], other Coleopterans (e.g., longhorn beetles) [30], [33], [34], and even Orthopterans [30] share similar colouration and patterning of some pachyrhynchid weevils, suggesting possible mimicry. Many predators show aversive responses towards harmless mimics of aposematic species, and examples are seen in diverse taxa such as coral snakes [35] and monarch butterflies [36].

We experimentally investigated the biological function and adaptive significance of conspicuous colouration in pachyrhynchid weevils. First, we tested the aposematic function of bright patterns by comparing responses of predators to weevils with the conspicuous colouration intact or experimentally masked. Next, we tested for geographic variation in predator responses to conspicuously coloured weevils by comparing the frequency of predatory attacks between allopatric and sympatric predator populations. Because individual weevil species composition can vary among islands, widespread predator species may overlap with different weevil species throughout their range, and some predator populations may not be exposed to weevils at all. We predicted that allopatric predators would show higher predation rates upon weevils because they lack prior experience with these prey. Finally, we studied whether allopatric predators can learn to avoid weevils after their first encounter, and their ability to retain any avoidance response over time. Our controlled experiments with using predator populations of different origins provide a powerful test of these predictions.

## Materials and Methods

### Ethics Statement

All work was conducted under animal ethics protocols of the Taiwanese Wildlife Conservation Act, governed by the Forestry Bureau, Council of Agriculture, Taiwan. This study was approved by the Taiwanese National Museum of Natural Science Animal Care and Use Committee (Protocol Permit NMNSHP12-001). After completing our experiments, we released all lizards and remaining weevils at their exact capture location. We did not observe any ill effects from lizards attempting to ingest, or successfully ingesting, weevils.

### Study Species

The weevil *Pachyrhynchus tobafolius* (Fig. 1a) is distributed on Green (22°39′33″N, 121°29′15E) and Orchid Islands (22° 3′18″N, 121°32′41″E), located 30 and 60 kilometres from southeastern Taiwan, respectively. The weevil *Kashotonus multipunctatus* (Fig. 1b) is endemic to Green Island. Neither of these species occurs on Taiwan. Both species have black bodies decorated with metallic green (*P. tobafolius*) or blue (*K. multipunctatus*) spots on dorsal surfaces of the head, thorax, elytra and legs (Fig. 1). *P. tobafolius* is the most abundant of the six pachyrhynchid weevil species on Orchid Island, whereas *K. multipunctatus* is the most abundant of the six species on Green Island [34]. Both *P. tobafolius* and *K. multipunctatus* are monomorphic (Fig. 1).

**Figure 2:**
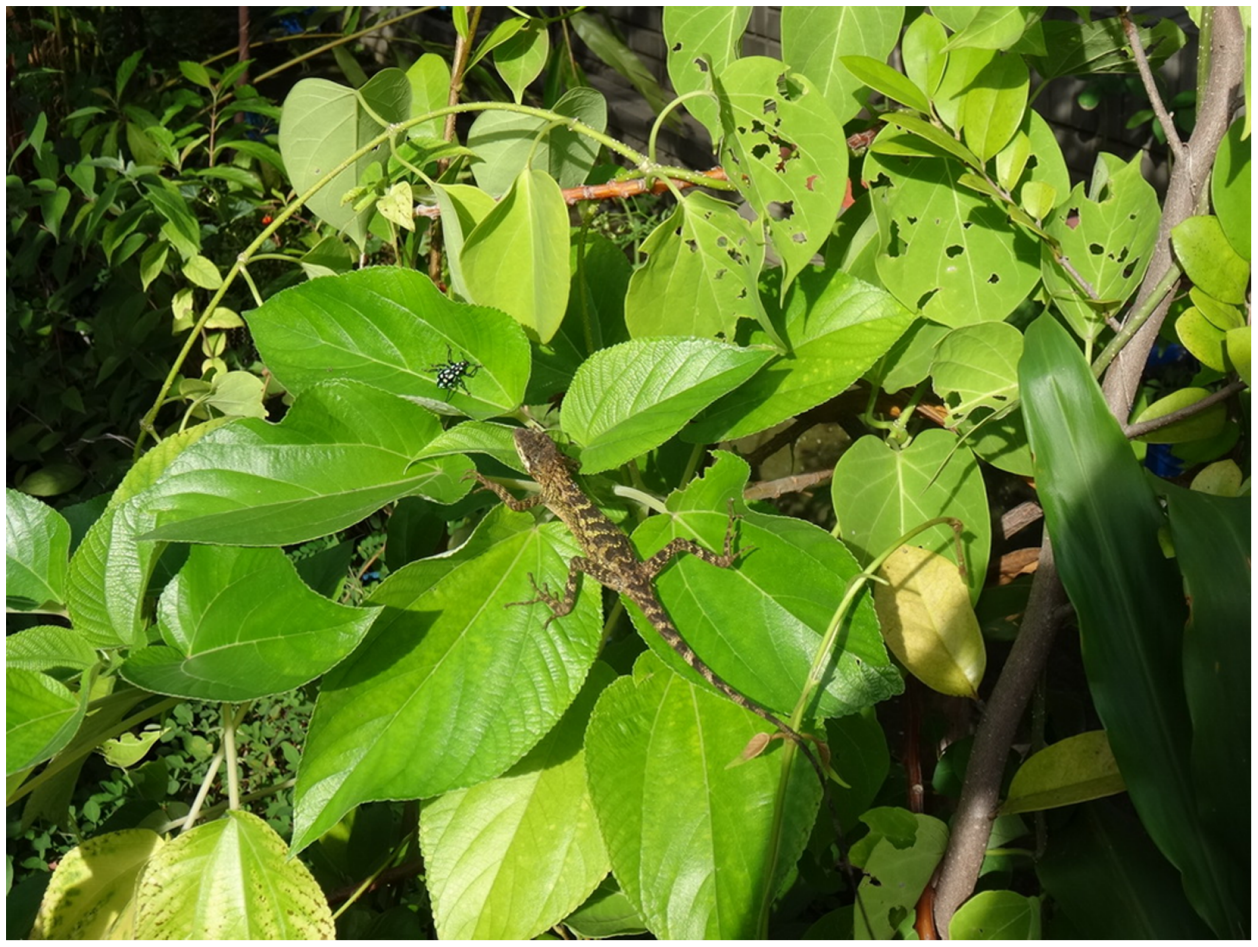
Swinhoe’s tree lizards (*Japalura swinhonis*) often inhabit the same trees with pachyrhynchid weevils, but they rarely attack weevils.

We tested the responses of visual predators to these two weevil species using predators collected from populations that were allopatric or sympatric with the weevils. Swinhoe’s tree lizards (*Japalura swinhonis*, Agamidae) are widespread, semi-arboreal predators of weevils that are often observed in weevil host plants alongside weevils [37] (Fig. 2). The average life span of this predator is likely three years (Y.-T. Lin, unpublished data). Like other ambush-foraging agamid lizards, this species uses visual cues to detect and capture prey [38]–[41].

### Study Populations

We collected weevils by hand or using an insect net from three sites on both Green and Orchid Islands. Weevils were maintained in plastic containers (19 cm diameter×5.5 cm height) in the laboratory under temperatures ranging from 20 to 25°C, and supplied once every three days with fresh leaves of their respective host plant (*P. tobafolius*: dalunot, *Pipturus arborescens* Urticaceae; *K. multipunctatus*: beach naupaka, *Scaevola taccada* Goodeniaceae). We captured lizards using a noose or by hand from populations on Green and Orchid Islands, as well as Jinshan in northern Taiwan (25°13′18.74″N, 121°38′10.55″E) and Kenting in southern Taiwan (22°1′5.64″N, 120°44′42.36″E; June 2011 - October 2012). We collected 156, 338, 178 and 126 lizards that were large enough to consume weevils from Orchid Island, Green Island, southern Taiwan, and northern Taiwan, respectively (n = 798 lizards). Lizards were transported to the laboratory in mesh bags, where each was assigned a unique identification number and housed individually in a plastic container (34×17×24 cm length width height). Water was available *ad libitum* and mealworms (*Tenebrio molitor*) were provided every three days. To help ensure that lizards were hungry at the time of testing, we did not feed them for the 24 hours preceding trials.

### Manipulation of Weevil Colour Pattern

We randomly assigned individual *P. tobafolius* and *K. multipunctatus* into two groups: a control group, composed of weevils with intact colour markings, and an experimental group, in which we masked all bright weevil colouration using a black marker pen (No. 3102003A, Simbalion). We also applied the mask to the black thoraces and elytra of control weevils (Fig. 1a, b). To confirm that our mask was biologically meaningful, we measured the reflectance spectra of different components of intact weevil patterns and the background colour of the weevil’s body when intact and masked (see Methods S1 for full methods and Figure S1 for the results).

### Experimental Design

Our experimental design focuses on two weevil species with intact or experimentally masked patterns, and lizards from three populations: (1) Taiwan lizards are allopatric to both weevil species, and no other pachyrhynchid weevils are found there; (2) Green Island lizards are sympatric with the weevils *P. tobafolius* and *K. multipunctatus*; and (3) Orchid Island lizards are sympatric with the weevil *P. tobafolius* and allopatric to the weevil *K. multipunctatus*. Our experiment was divided into two parts: (1) the function of colourful markings on weevils, and (2) geographic variation of predatory responses toward weevils. In the first part, lizards from Orchid and Green Island were used to compare the responses of lizards toward intact and masked weevils. In the second part of our study, we compare the predatory responses between sympatric and allopatric predator populations toward intact weevils. We predicted that allopatric predators would show higher predation rates upon weevils than sympatric predators, because they lack prior experience with these prey. By contrast, we predicted that sympatric predators would be more likely to consume masked weevils than those with bright colouration, because of prior negative experience with these prey. We used chi-squared tests to determine whether sympatric lizard populations differed significantly in their behavioural responses towards intact and masked weevils (Orchid Island vs. *P. tobafolius*; Green Island vs. *P. tobafolius* and *K. multipunctatus*), and whether responses toward intact weevils were similar between sympatric (*P. tobafolius* vs. Orchid and Green Island; *K. multipunctatus* vs. Green Island) and allopatric (Taiwan) predator populations.

### Behavioural Trials

Our behavioural trials were conducted at room temperature (25–31°C) between 9∶00 AM and 5∶00 PM, when lizards were active. For each trial, we placed a lizard into an arena (39×30×20 cm length width height) for one minute prior to introducing a weevil, which was tied to a black cotton thread and positioned approximately 10 cm in front of the lizard. Trials were terminated after two minutes because most lizards attacked prey within this time. The behavioural response of each lizard was recorded as “attack” or “ignore.” Attack behaviour was defined as a lizard approaching and biting the weevil, which was either consumed or spat out. Ignore behaviour was defined as a lizard not attacking the weevil during a two-minute trail. Immediately after each weevil trail, we tested whether lizards were hungry by offering each a mealworm tied to a cotton thread. If the lizard ate the mealworm, we classified it as having ignored the weevil as palatable prey. We excluded the individuals that neither attacked the weevil nor consumed the mealworm from analysis because these lizards may not have been hungry during testing.

### Learning and Memory Retention

We used the lizard populations from Taiwan to test whether allopatric lizards can learn to avoid *K. multipunctatus* weevils after their first encounter, and whether any avoidance behaviour is retained over time. Allopatric lizards from Taiwan that successfully attacked a weevil were randomly assigned to one of four groups, which were presented with another weevil after 1, 5, 13, or 23 days. Between initial and subsequent exposure to weevils, lizards were fed mealworms every three days until 24 hours before the second trial. We used a contingency table analysis to test whether the frequency of predatory attacks differed with the time interval passing before being offered another weevil. In the learning and memory retention trials, each lizard was tested once initially and again at the assigned interval (1, 5, 13, or 23 days), and thus all comparisons across intervals are independent.

## Results

Among the 798 individual lizards tested, most attempted to ingest the mealworm after being offered a weevil; we excluded instances in which neither was consumed (2.6–6.3% of individuals from each population; n = 4, 15, 9 and 8 individuals, respectively).

### Predatory Responses Towards Intact and Masked *P. tobafolius*

**Figure 3:**
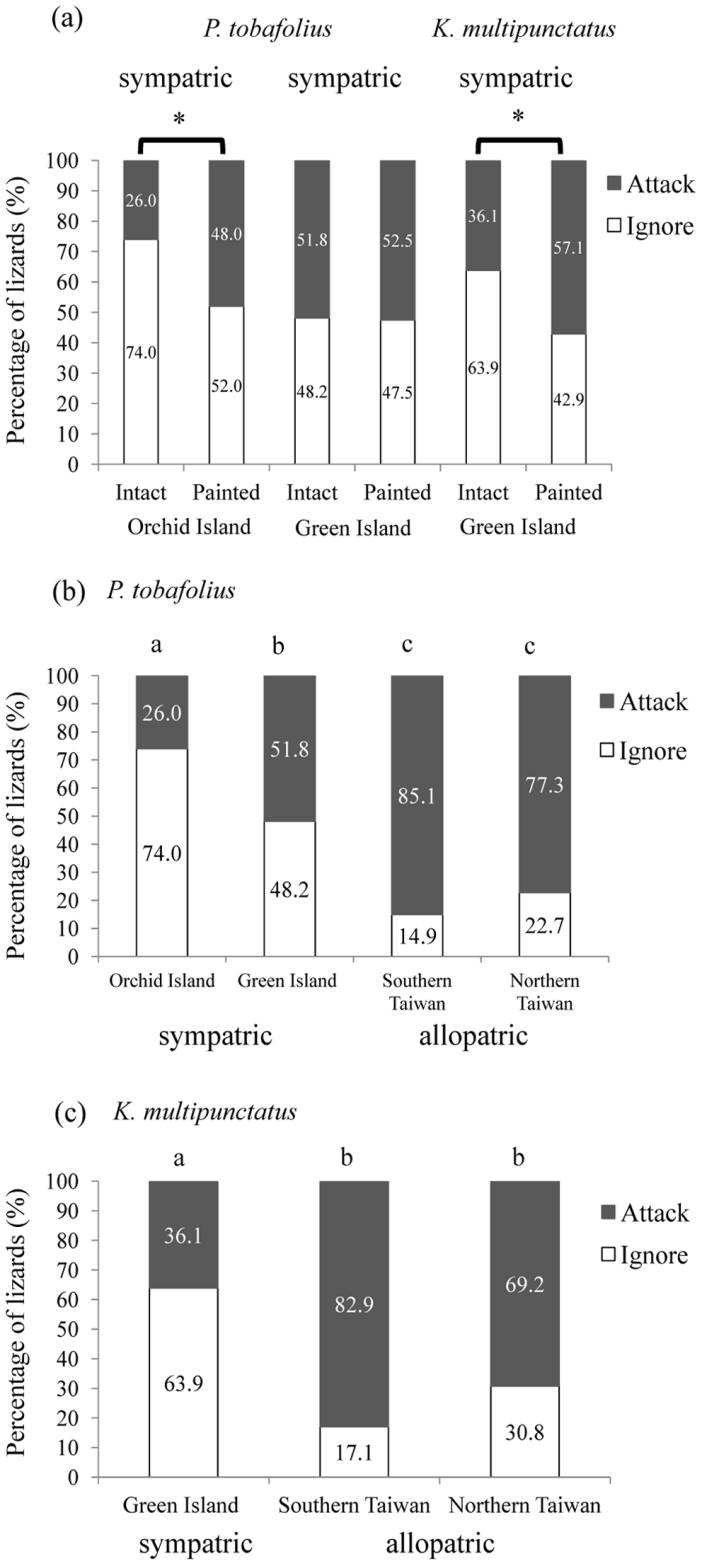
The percentage of Swinhoe’s tree lizards (*Japalura swinhonis*) that exhibited different predatory behaviour, shown for sympatric and allopatric predator populations. (a) The first four bars represent the response of sympatric lizards from Orchid Island and Green Island to *P. tobafolius*. The rightmost bars show the response of sympatric lizards from Green Islands to *K. multipunctatus*. Asterisk represents significant difference (*P*<0.01); (b) Response of lizards from different localities to *P. tobafolius* with intact markings; (c) Response of lizards from different localities to *K. multipunctatus* with intact markings. Different letters indicate statistically significant differences. Note that *K. multipunctatus* does not occur on Orchid Island.

For the sympatric lizards from Orchid Island, only 26.0% (20/77) attacked intact *P. tobafolius*, whereas a significantly higher percentage of the lizards 48.0% (36/75) attacked masked weevils (χ^2^ = 7.92, df = 1, *P*<0.01) (Fig. 3a). However, sympatric lizards from Green Island exhibited similar attack rates towards intact (51.8%, 43/83) and masked (52.5%, 42/80) *P. tobafolius* (χ^2^ = 0.01, df = 1, *P = *0.92) (Fig. 3a).

### Predatory Responses Towards Intact and Masked *K. multipunctatus*

The sympatric lizards from Green island had significantly higher attack rates towards masked (57.1%, 44/77) rather than intact (36.1%, 30/83) *K. multipunctatus* (χ^2^ = 7.08, df = 1, *P*<0.01). The attack rates of lizards upon masked weevils were similar between Orchid Island lizards for *P. tobafolius* and Green Island lizards for *K. multipunctatus* (Fig. 3a).

### Predatory Responses of Sympatric and Allopatric Lizards Toward *P. tobafolius*

The allopatric lizards from southern (n = 87) and northern (n = 66) Taiwan and the sympatric lizards from Green Island (n = 83) showed significantly higher attack rates towards *P. tobafolius* than did sympatric lizards from Orchid Island (n = 77) (southern Taiwan vs. Orchid Island, χ^2^ = 58.29, df = 1, *P*<0.01; northern Taiwan vs. Orchid Island, χ^2^ = 37.41, df = 1, *P*<0.01; Green Island vs. Orchid Island, χ^2^ = 11.17, df = 1, *P*<0.01) (Fig. 3b). We found similar predatory responses in the two allopatric lizards from southern (n = 82) and northern Taiwan (n = 52).

### Predatory Responses of Sympatric and Allopatric Lizards Toward *K. multipunctatus*

These allopatric populations also had significantly higher attack rates upon *K. multipunctatus* than did sympatric lizards from Green Island (n = 83) (southern Taiwan vs. Green Island, χ^2^ = 37.43, df = 1, *P*<0.01; northern Taiwan vs. Green Island, χ^2^ = 14.01, df = 1, *P*<0.01) (Fig. 3c). Overall, the vast majority of lizards that attacked the weevils after initially biting them spat them out (97.1%), with only a few lizards chewing up, crushing, or consuming weevils (2.9%; n = 13/444 of the lizards that attacked weevils).

### Memory Retention of Prey Avoidance

**Figure 4:**
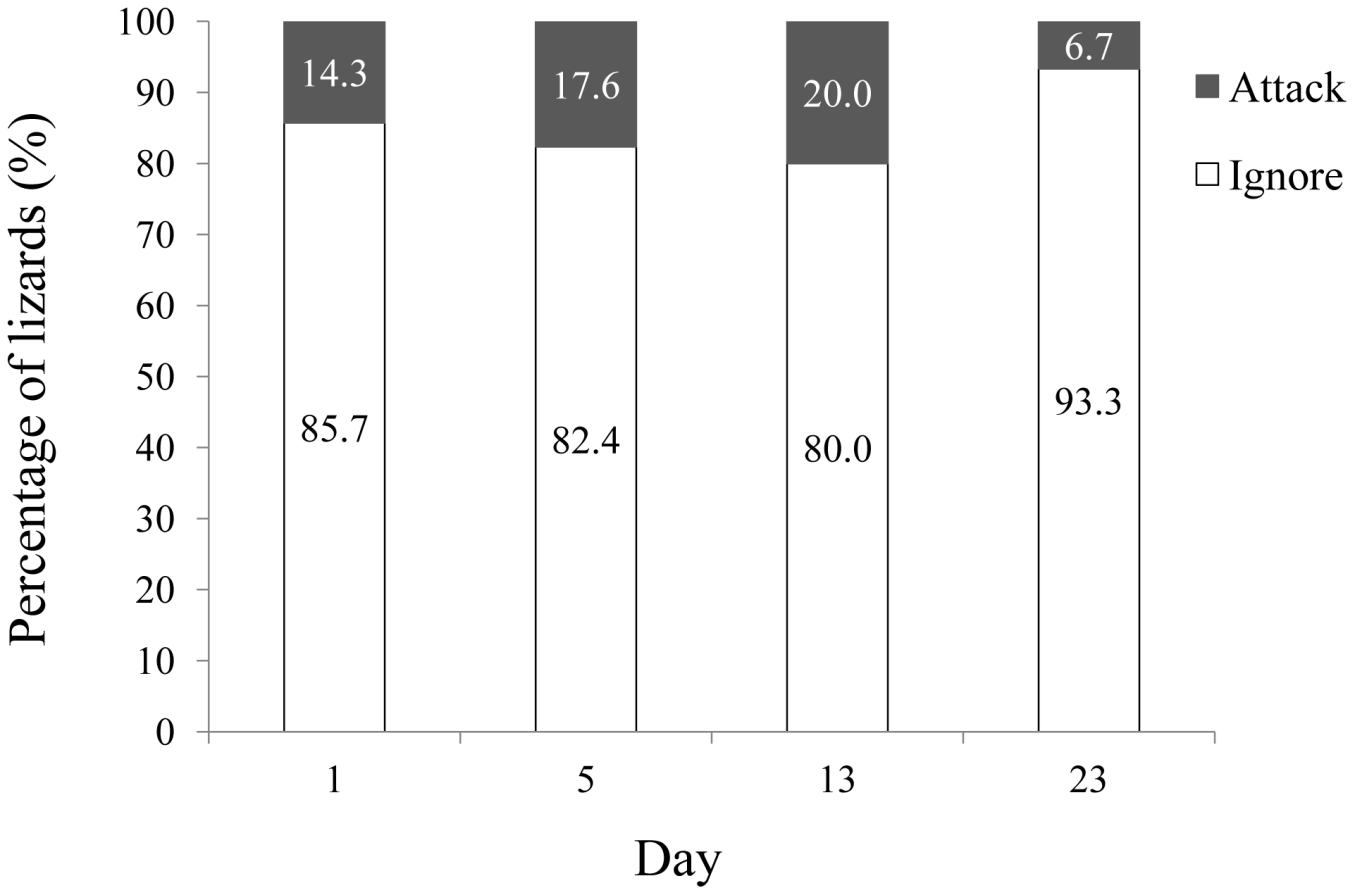
Memory retention tests of the predatory responses of allopatric Swinhoe’s tree lizards (*Japalura swinhonis*) to colourful weevil prey (*K. multipunctatus*).

After initially attacking a weevil, allopatric lizards strongly avoided consuming a subsequent weevil for up to 23 days (Fig. 4). We found no significant difference among treatments in the propensity of individual lizards to re-attack weevils after different time intervals (χ^2^ = 1.22, df = 3, *P = *0.748). After a single day, only 14.3% of lizards were willing to attack a weevil, and this strong avoidance behaviour was maintained for at least 23 days after initially attacking a weevil (6.7% attack rate; Fig. 4).

## Discussion

Our study provides powerful experimental evidence that the conspicuous colouration of pachyrhynchid weevils can function as aposematic signals that deter attacks by sympatric predators. The colour markings of *P. tobafolius* and *K. multipunctatus* greatly decreased attack rates of sympatric Swinhoe’s tree lizards from Orchid and Green Islands, respectively. Although allopatric predators were more likely to attack weevils with intact colouration, after a single encounter lizards rapidly learned to avoid weevils for more than 23 days. These types of (presumably) learned avoidance of prey by predators can lead to effective mimicry of the aposematic signal by other taxa. This provides a striking example of how aposematism can function in nature, and is consistent with the responses of other predators towards organisms with conspicuous colouration [6], [7], [20], [42]–[45]. The Old World tropics supports a diverse array of pachyrhynchid weevils, along with other diverse taxa that are visually similar, including other weevil genera [27] and other insect families (e.g., Coleopterans, Orthopterans [30], [33], [34]). These taxa may benefit from reduced predation by mimicking the colouration of aposematic weevils.

To aid in predator deterrence, many animals displaying aposematic signals emit or possess chemical compounds, such as alkaloids [46], cardenolides [47], or formic acid [48]. Earlier studies have been unable to find chemical defences or secretory organs in pachyrhynchid weevils [27], [31]. The primary defensive mechanism of these weevils is likely to be their tough exoskeleton [29], [30]. Of the hundreds of lizards that attacked weevils in our study, only a few individuals were willing to consume them; instead, most lizards spat the weevil out almost immediately. The evolution of aposematism is always controversial because conspicuous colouration is assumed to have selective disadvantages, and thus prey can be detected and noticed more easily, which should reduce fitness. However, if the predator can learn and memorise the connection between warning colouration and unprofitability, aposematism can become established [4]. In our study system, the tough exoskeleton may provide efficient defence for weevils, and facilitate the evolution of conspicuous colouration on pachyrhynchid weevils.

The variation in responses of the sympatric lizards to these weevils may depend upon prior experience by individual predators, which can be influenced by geographic and habitat overlap, and local abundance [49]. For example, the sympatric lizards from Green Island attacked both intact and masked *P. tobafolius* to similar degrees. On Green Island, *P. tobafolius* is less abundant than *K. multipunctatus*, likely because of low host plant abundance [34]. When prey abundance decreases, predators such as these lizards may be less likely to have experienced some aposematic patterns, and consequently may mistakenly attack unpalatable prey. These types of “mistakes” may lead to selection for the ability to recognize other attributes that co-evolve with aposematic signals, such as body size or shape. This leads to a more general recognition of prey by predators, likely enhancing fitness.

Overall, the attack rates of sympatric lizards were significantly higher for masked weevils than attack rates for intact weevils, but these rates were lower than those of allopatric lizards attacking intact weevils (Fig. 3). These results suggest that lizards can recognise the unprofitability of masked weevils using cues other than bright colouration. Colour is only one component of an aposematic signal, which can include complex patterns [9], [50], shapes [6], [7], [51], [52], sizes [53], and even odours [54]. In our study, almost half of all sympatric lizards avoided attacking masked weevils (42.9–52.0%, depending on the population), suggesting that physical shape, size, behaviour, or scent can provide additional cues that are readily avoided in the absence of the aposematic signal.

The results of our study provide the first demonstration of geographic variation in prey recognition by a squamate reptile. Similar responses have been reported in other taxa, especially avian predators [43], [55]. Geographic variation of prey recognition may result from different local prey communities, which can impose diverse selection pressures upon predators even in adjacent geographic regions [43], [56]. Allopatric lizards were much more likely to attack aposematic weevils, probably because they had no prior experience with the patterns from these prey species. This is a common phenomenon in many predator-prey systems [43], [55], [56]. For example, poison frogs show strong geographic variation in colouration and local predators can better recognize local aposematic forms than other geographically distant forms [43], [55]. In some cases, aposematic species do not show local variation in colour or patterning, and consequently local predators prefer novel, unfamiliar phenotypes to the local aposematic form [20], [55]. In other instances, however, these predators may avoid novel aposematic prey because of neophobia or dietary conservatism [4], [18], [57].

A substantial proportion of the sympatric lizards we tested consumed their respective local weevil species. Incomplete avoidance behaviour by predators represents an ongoing learning/continued testing process [55], poor learning/forgetting [58], or predator naivety [59]. Many organisms, especially birds, can learn to avoid unprofitable prey based on novel colour signals [8], [19], [21]. However, studies focused on avoidance learning in reptiles remain rare [24]. Swinhoe’s tree lizards can successfully learn to avoid weevils immediately after only a single encounter, and retain this avoidance behaviour for at least 23 days. In many studies of avian predators, the experimental duration of memory retention tests is less than seven days after the first treatment [8], [19]. Even in memory tests of garter snakes (*Thamnophis radix*) that continued for 22 days, attack latencies towards aposematic prey decreased over time [24]. By contrast, Swinhoe’s tree lizards maintained high and stable rates of continued prey avoidance behaviour (Fig. 4). Because the ability to recognize unpalatable prey did not decline by the end of our 23-day trial, the aposematic signals of these weevils not only influence predatory responses, but also help predators form strong and long-lasting memory associations.

## Supporting Information

Figure S1 **Results of reflectance spectra readings.** Reflectance spectra of the dark background colour of weevils, the bright patterning of weevils, and the black marker used to mask the colourful patterning for (a) *Pachyrrhynchus tobafolius* and (b) *Kashotonus multipunctatus*. Note that the black marker more closely matches the background colouration of both weevil species than the bright patterns that we masked.
(TIF)
