# Supplementary figures and images for: The Functional Significance of Aposematic Signals: Geographic Variation in the Responses of Widespread Lizard Predators to Colourful Invertebrate Prey
Source: PLoS One. 2014 Mar 10;9(3):e91777. doi: 10.1371/journal.pone.0091777 (PMC3948897; doi:10.1371/journal.pone.0091777)

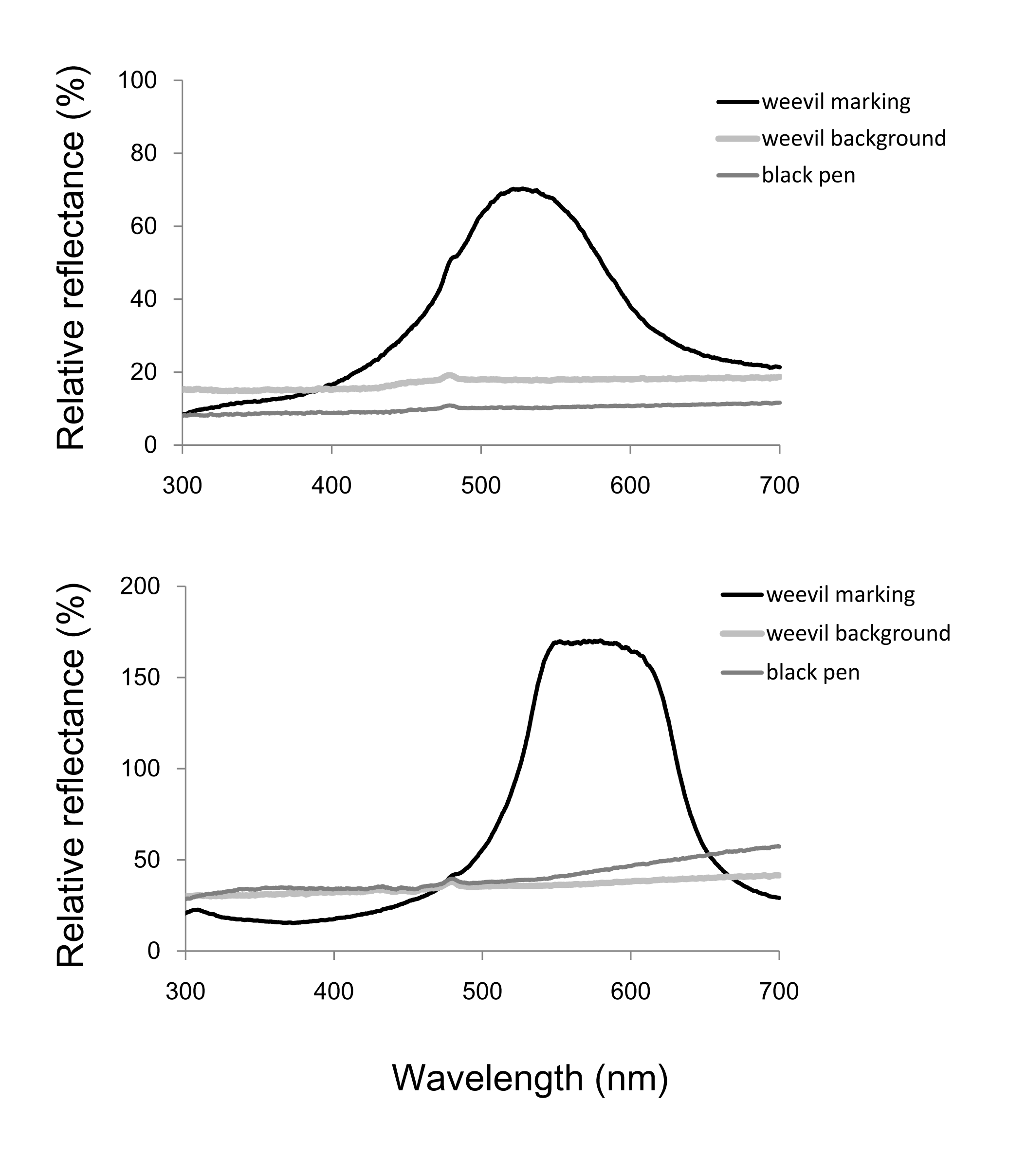

Supplement: Figure S1 — Results of reflectance spectra readings. Reflectance spectra of the dark background colour of weevils, the bright patterning of weevils, and the black marker used to mask the colourful patterning for (a) Pachyrrhynchus tobafolius and (b) Kashotonus multipunctatus. Note that the black marker more closely matches the background colouration of both weevil species than the bright patterns that we masked. (TIF) [file pone.0091777.s001.tif]
